# Efficacy and Safety of Zolpidem for Focal Dystonia After Neurosurgical Treatments: A Retrospective Cohort Study

**DOI:** 10.3389/fneur.2022.837023

**Published:** 2022-05-03

**Authors:** Shiro Horisawa, Kotaro Kohara, Hiroki Ebise, Masahiko Nishitani, Takakazu Kawamata, Takaomi Taira

**Affiliations:** Department of Neurosurgery, Tokyo Women's Medical University, Tokyo, Japan

**Keywords:** dystonia, efficacy, neurosurgery, safety, zolpidem

## Abstract

Although there are several reports of the significant efficacy of zolpidem for treating dystonia, zolpidem is still considered an anecdotal treatment. Here, we evaluated the efficacy and safety of zolpidem for treating residual dystonia in patients who previously received various neurosurgical treatments majorly including deep brain stimulation and radiofrequency ablation. We retrospectively reviewed medical records from January 2021 to September 2021 to identify patients with dystonia who had been prescribed zolpidem after undergoing neurosurgery. Twenty patients were enrolled in this study, including those with blepharospasm (two), tongue dystonia (four), mouth dystonia (one), spasmodic dysphonia (two), cervical dystonia (six), focal hand dystonia (three), hemidystonia (two), blepharospasm with cervical dystonia (one), and mouth dystonia with cervical dystonia (one). Single doses of zolpidem ranged between 2.5 and 10 mg, while daily dosages ranged from 10 to 30 mg. The zolpidem dose prescribed was 5–10 mg, with single and daily doses of 7 ± 2.9 and 14.5 ± 6.0 mg, respectively. With zolpidem administration, the participants' Burke-Fahn-Marsden Dystonia Rating Scale-Movement Scale score significantly improved from 8.1 ± 6.7 to 3.7 ± 2.5 (50.6% improvement, *p* < 0.0001). Improvements in arm dystonia, blepharospasm, and spasmodic dysphonia were observed using the Arm Dystonia Disability Scale, Jankovic Rating Scale, and Voice Handicap Index, respectively. No improvements were observed in cervical dystonia on the Toronto Western Spasmodic Torticollis Rating Scale. Drowsiness, including three cases each of mild and moderate drowsiness, was the most frequent adverse effect (30%), which persisted for 2–3 h. Transient amnesia and rapid eye movement sleep behavior disorder occurred in two patients and one patient, respectively. Although our findings suggest that zolpidem can be a valuable treatment option for patients with residual dystonia after neurosurgical treatments, the beneficial effects for cervical dystonia were limited.

## Introduction

Treatments for dystonia include pharmacological therapies, botulinum toxin injections, and surgery (1). Botulinum toxin injection and surgical treatment are widely accepted treatments for medically refractory dystonia (2, 3). Pharmacological therapy with trihexyphenidyl, clonazepam, baclofen, and dopamine-related medications is generally the first-line treatment for dystonia (2).

Zolpidem is an imidazopyridine, non-benzodiazepine hypnotic agent that has been widely prescribed for the treatment of insomnia. Zolpidem was reported to have therapeutic effects on Parkinson's disease, which was confirmed by several studies including a double-blinded, placebo-controlled study. Additionally, several reports have shown significant efficacy of zolpidem for dystonia (4–13). However, zolpidem remains an anecdotal treatment due to the lack of randomized and controlled studies, and the mechanism of action of zolpidem in relieving dystonia remains unclear.

We have performed several neurosurgical treatments for dystonia, including selective peripheral denervation, deep brain stimulation (DBS), and ablative surgeries using radiofrequency, gamma knife, and focused ultrasound (14–19). All surgical candidates in those reports were refractory to conventional oral medications including trihexyphenidyl, clonazepam, and baclofen. Because botulinum toxin injections are covered by health insurance for the treatment of cervical dystonia and blepharospasm, many dystonia patients other than cervical dystonia and blepharospasm in Japan cannot afford it. We prescribed 5–10 mg of zolpidem at a time to postoperative neurosurgical patients with dystonia. In this study, we report the efficacy and safety of zolpidem for treating residual dystonia in patients who previously received neurosurgical treatment.

## Materials and Methods

### Patient Population

We retrospectively reviewed medical records from January 2021 to September 2021 to identify patients with dystonia who were prescribed zolpidem. Zolpidem was prescribed only to patients who were not satisfied with surgical outcomes and wanted further improvements. We included patients with dystonia who had received zolpidem for dystonia treatment. The exclusion criteria were missing data regarding subjective evaluation using scales or side effects and concomitant use of additional medications or botulinum toxin injections after starting zolpidem use.

### Medication

Zolpidem was prescribed at a single dose of 5 mg daily for the first 3 days. Thereafter, the dose was increased to a single dose of 10 mg daily tolerated. In case of difficulty in continuation of zolpidem due to its side effects, such as drowsiness, the dose was decreased to a single dose of 2.5 mg per time. Patients were allowed to take zolpidem several times per day if the daily dose was <10 mg.

### Evaluation

The patients' demographic and clinical characteristics, including the distribution and etiology of dystonia, failed treatments, including surgery for dystonia prior to zolpidem prescription, zolpidem dose, the effective duration of treatment, and adverse events were evaluated. Patients receiving DBS were evaluated with stimulating-on conditions.

All patients were evaluated 1 month after starting zolpidem use at the outpatient clinic. Previous studies reported that onset of effects and peak effects after zolpidem administration were 15–45 min and 1–2 h, respectively (5, 7–10, 12). Therefore, evaluation was performed before the initiation of zolpidem and at the time of maximum drug concentration (1–2 h after oral administration). Rating scales were used for evaluation. All the patients were administered the Burke-Fahn-Marsden Dystonia Rating Scale (BFMDRS)-Movement Scale (BFMDRS-MS; range: 0–120; higher scores indicate greater severity). Patients with blepharospasm were administered the Jankovic Rating Scale (JRS; range: 0–8; higher scores indicate greater severity), and those with spasmodic dysphonia were administered the Voice Handicap Index (VHI; range: 0–120; higher scores indicate greater voice-related handicap). Patients with cervical dystonia were administered the Toronto Western Spasmodic Torticollis Rating Scale (TWSTRS; range: 0–85; higher scores indicate greater severity, disability, and pain), whereas those with focal hand dystonia were administered the Arm Dystonia Disability Scale (ADDS; range: 0–100%; lower scores indicate greater disability).

### Statistical Analysis

Statistical analysis was performed using the JMP statistical package, version 15.0.0 (SAS Institute, Cary, NC). The data were considered non-parametric; therefore, the Wilcoxon signed-rank test was used to compare the pre- and post-treatment BFMDRS-MS scores. Statistical significance was set at *p* < 0.05.

### Ethical Considerations

The data for this study were retrospectively collected and analyzed. The Ethics Committee of our institution approved this study, and considering the observational nature of the study, the requirement for the provision of consent by patients was waived. Written informed consent for the publication of the videos was obtained.

## Results

Twenty-seven patients were prescribed zolpidem for dystonia treatment. Seven patients were excluded, three due to missing data regarding subjective evaluation scales and four due to concomitant use of additional medications or botulinum toxin injections after starting zolpidem use. The patients' characteristics are shown in Table 1. Twenty patients were enrolled in this study, including those with blepharospasm (two), tongue dystonia (four), mouth dystonia (one), spasmodic dysphonia (two), cervical dystonia (six), focal hand dystonia (three), hemidystonia (one), focal hand and foot dystonia (one), blepharospasm with cervical dystonia (one), and mouth dystonia with cervical dystonia (one). The etiologies of dystonia were idiopathic in 13 patients, post-stroke in four patients, post-traumatic in one patient, drug-induced (tardive) in one patient, and hereditary (DYT-1 dystonia) in one patient. Failed treatments prior to surgery included botulinum toxin injections (15 patients), trihexyphenidyl (1–6 mg/day, 18 patients), clonazepam (1.5 mg/day, 10 patients), and baclofen (15 mg/day, two patients). The neurosurgical treatments performed in the patients included deep brain stimulation (16 patients), radiofrequency ablation (12 patients), gamma knife ablation (one patient), and selective peripheral denervation (one patient). The pre- and post-operative BMFDRS-MS scores were 11.1 ± 8.1 and 8.2 ± 6.6, respectively (25.8% improvement).

**Table 1:** Patient characteristics and clinical outcomes.

**Case**	**Distribution of dystonia**	**Etiology**	**Sex**	**Age at onset (years)**	**Disease duration (years)**	**Failed treatments prior to surgery**	**Neurosurgical treatments**	**Interval between last surgery and zolpidem administration**	**Zolpidem**	**BFMDRS-MS score**			
									**Single dose/Daily dose**	**Pre surgery**	**Pre medication**	**Post medication**	**% improvement**
1	Blepharospasm	Primary	Female	52	11	BTX, Tri, Clo	GPi-DBS*, GPi-RF	45	2.5/10 mg	8	8	4	50
2	Blepharospasm	Primary	Female	63	6	BTX	GPi-DBS	13	10/20 mg	12	8	4	50
3	Spasmodic dysphonia	Stroke	Male	23	6	BTX, Tri, Clo	GPi-DBS*	20	10/20 mg	4	4	2	50
4	Spasmodic dysphonia	Primary	Male	44	5	BTX, Tri	GPi-DBS*, GPi-RF	50	5/10 mg	6	3	2	33.3
5	Tongue dystonia	Primary	Female	59	10	BTX	FF-DBS	6	5/10 mg	4	2	0	100
6	Tongue dystonia	Primary	Male	29	7	BTX	FF-DBS, RF	6	10/20 mg	4	2	0.5	75
7	Tongue dystonia	Primary	Female	44	1	BTX, Tri	PTT-RF	3	5/10 mg	9	1	0.5	50
8	Tongue dystonia	Primary	Male	33	2	Tri	FF-DBS, PTT- RF	3	5/10 mg	4	1	0.5	50
9	Mouth dystonia	Primary	Male	73	2	BTX, Tri, Clo	GPi-DBS	12	2.5/10 mg	6	6	4	33.3
10	Blepharospasm/ Cervical dystonia^†^	Primary	Male	47	4	Tri	PTT-GK	3	5/10 mg	12	12	10	16.7
11	Mouth dystonia/ Cervical dystonia^†^	Tardive	Female	52	12	BTX, Tri, Bac	GPi-DBS	60	5/10 mg	12	10	8	20
12	Cervical dystonia^‡^	Primary	Male	52	13	Tri, Clo	GPi-DBS	70	5/10 mg	8	4	2	50
13	Cervical dystonia^‡^	Traumatic	Male	45	2	Tri, Clo, Bac	FF-DBS	6	10/10 mg	6	4.5	4.5	0
14	Cervical dystonia^†^	Tardive	Female	51	2	BTX, Tri, Clo	PTT-RF	3	5/10 mg	6	4.5	4.5	0
15	Cervical dystonia^‡^	Primary	Male	47	9	Tri, Clo	GPi-DBS, GPi-RF, SPD	12	5/10 mg	8	6	6	0
16	Focal hand dystonia	Stoke	Female	5	46	BTX, Tri	GPi-DBS*, Vo-RF	6	10/20 mg	16	16	6	62.5
17	Hemidystonia	Stroke	Male	28	2	BTX, Tri, Clo	DN-DBS**	6	10/30 mg	24	24	5	79.2
18	Focal hand dystonia	Stroke	Male	48	8	Tri, Clo	PTT-RF	12	10/20 mg	16	16	4	75
19	Hemidystonia	Primary	Male	27	9	BTX, Tri	DN-DBS, Vim-DBS**, Vo-RF,	16	10/20 mg	36	20	5	75
20	Focal hand dystonia	Hereditary (DYT-1)	Male	9	19	BTX, Tri	Vo-DBS, GPi-RF	10	10/20 mg	20	12	6	50

*BFMDRS-MS, Burke-Fahn-Marsden Dystonia Rating Scale-Movement Scale; BTX, botulinum toxin injections; Tri, trihexyphenidyl; Clo, clonazepam; Bac, baclofen; GPi, Globus pallidus internus; FF, Forel's field; PTT, Pallidothalamic tract; DN, Dentate nucleus; Vo, Ventro-oral nucleus; DBS, deep brain stimulation; RF, radiofrequency ablation; GK, gamma knife ablation; SPD, selective peripheral denervation.*

*^†^Phasic cervical dystonia.*

*^‡^Tonic cervical dystonia.*

*DBS was removed for insufficient efficacy.

**DBS was removed for infection.

The mean single and daily doses of zolpidem were 7 ± 2.9 and 14.5 ± 6.0 mg, respectively. The daily dose of zolpidem was spontaneously increased to 20–30 mg in eight patients to improve their quality of life. Among eight patients, five with focal hand dystonia, focal hand and foot dystonia, and hemidystonia (case 16–20) experienced pain due to severe dystonic postures. Both pain and dystonia were remarkably relieved by zolpidem administration. Case 2 was functionally blind due to severe blepharospasm (forced eyelid closure), and at least 10 mg of zolpidem twice a day had to be administered to sustain eye-opening. Case 3 with spasmodic dysphonia and Case 6 with tongue dystonia had difficulty with daily communication with others due to dystonia. A single dose of 10 mg of zolpidem twice a day was necessary for their daily lives.

**Figure 1:**
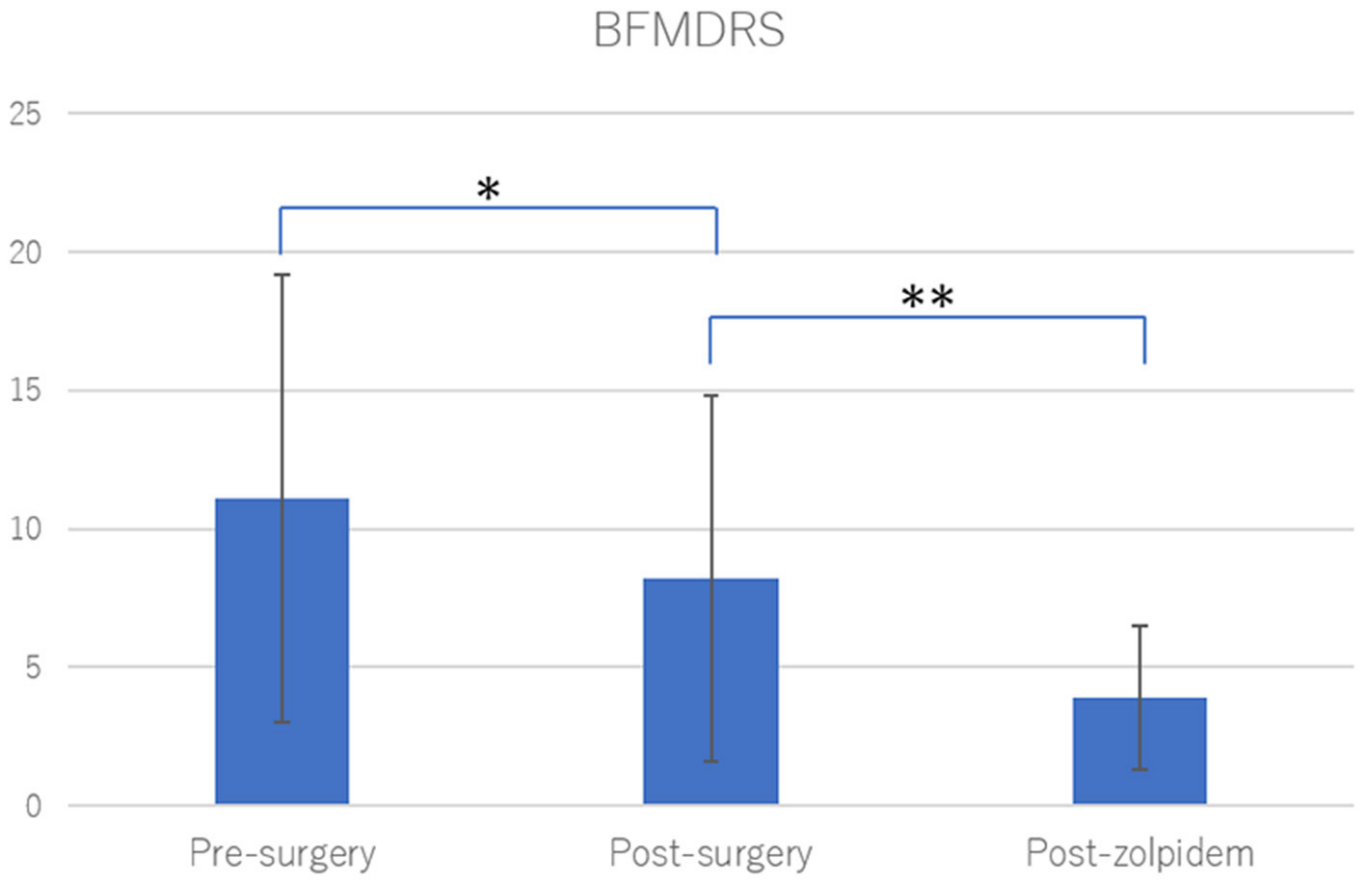
Burke-Fahn-Marsden Dystonia Rating Scale (BFMDRS) after neurosurgical treatments and zolpidem. Neurosurgical treatments significantly improved dystonia among the study participants (29.9% improvement, *p = 0.0002). Zolpidem administration significantly improved residual dystonic symptoms after neurosurgical treatments (50.6% improvement, **p < 0.0001). Statistical significance was evaluated by Wilcoxon signed rank test.

**Figure 2:**
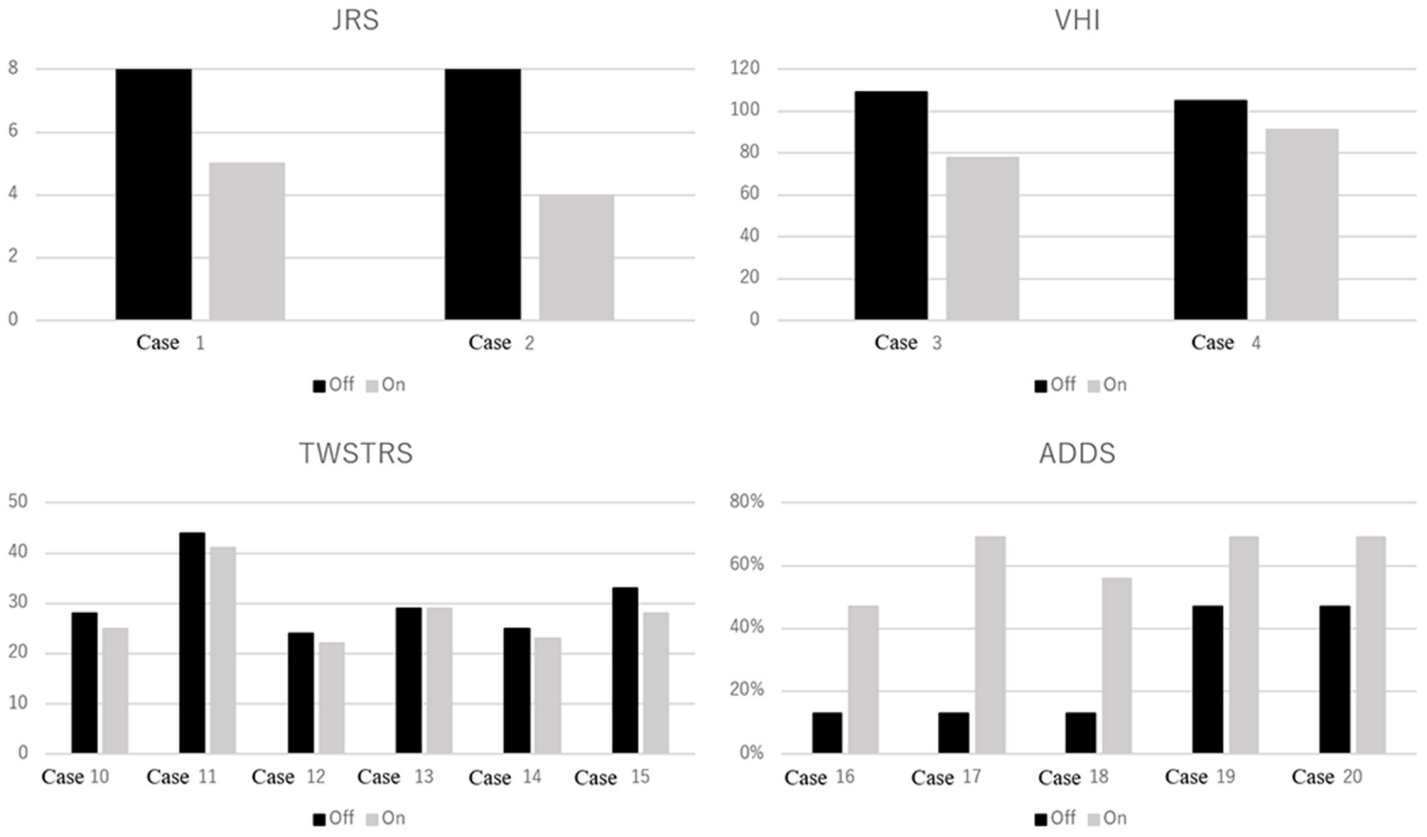
Focal dystonia rating scale scores with and without medication. JRS, Jankovic Rating Scale. Range: 0–8. Higher scores indicate greater blepharospasm severity. VHI, Voice Handicap Index. Range: 0–120. Higher scores indicate greater voice-related handicap in patients with spasmodic dysphonia. TWSTRS, Toronto Western Spasmodic Torticollis Rating Scale. Range: 0–85. Higher scores indicate greater severity, disability, and pain in patients with cervical dystonia. ADDS, Arm Dystonia Disability Scale. Range: 0–100%. Lower scores indicate greater disability in patients with focal hand dystonia.

With zolpidem administration, the BFMDRS-MS score significantly improved from 8.1 ± 6.7 to 3.7 ± 2.5 (50.6% improvement, *p* < 0.0001; Figure 1, Table 2). Three patients with cervical dystonia did not respond to zolpidem, while 13 patients (68.4%) showed 50 to 100% reduction in BFMDRS-MS score. Six patients with cervical dystonia responded poorly to zolpidem in our study. The mean total TWSTRS score was 30.5 ± 7.3 before administration and 28 ± 6.9 after zolpidem administration (5.8 ± 2.0 mg). Although severity and disability scores did not change after zolpidem administration, pain scores decreased from 8.3 ± 3.8 to 6.1 ± 2.7 with zolpidem administration. The mean BFMDRS-MS neck subscale score was 5 ± 0.9 before administration and 4.6 ± 1.6 after administration (8% improvement). Symptomatic improvements were confirmed in patients with focal hand dystonia, blepharospasm, and spasmodic dysphonia using the ADDS, JRS, and VHI, respectively; however, no improvements in TWSTRS score were noted in patients with cervical dystonia (Figure 2). The representative movies illustrating spasmodic dysphonia (case 4), tongue dystonia (case 5), and focal hand dystonia (case 16) are shown in Supplementary Videos 1–3.

**Table 2:** BFMDRS scores off and on medication.

**BFMDRS-MS**	**Number of patients**	**Off medication**	**On medication**	
Total	20	8.2 ± 6.6	3.9 ± 2.6	*p < 0.0001
Eyes	3	7.3 ± 1.2	4	
Mouth	6	2.7 ± 2.0	1.3 ± 1.5	
Speech/swallowing	2	3.5 ± 0.7	2	
Neck	6	5.2 ± 0.9	4.8 ± 1.6	
Arm	5	15.2 ± 1.8	4.8 ± 1.1	
Leg	2	6 ± 2.8	1	

*BFMDRS-MS: Burke-Fahn-Marsden Dystonia Rating Scale-Movement Scale.*

*The Wilcoxon signed-rank test was used to compare the BFMDRS-MS scores off and on medication.

The duration of beneficial effects of zolpidem on dystonias ranged from 3 to 4 h, and the time-to-effect ranged from 15 to 30 min, which corresponded to previous study findings (5, 7–10, 12). Drowsiness, including three cases each of mild and moderate drowsiness, was the most frequent adverse effect (30%), which persisted for 2–3 h. Transient amnesia and rapid eye movement sleep behavior disorder occurred in two patients and one patient, respectively. Four patients with hand dystonia, excluding those with DYT-1 dystonia, had a fixed dystonic posture (clenched fist). Five milligrams of zolpidem did not improve hand dystonia in these four patients; however, 10 mg significantly improved their symptoms. Two of these patients increased the single dose of zolpidem to 15–20 mg once on their own and showed significant dose-dependent improvement. However, sleepiness increased with increasing doses; therefore, zolpidem was discontinued. Two patients with cervical dystonia who did not achieve any benefit ceased taking zolpidem voluntarily. One patient with blepharospasm and one patient with mouth dystonia showed significant improvement without any complications with 2.5 mg of zolpidem. Eighteen patients thought that zolpidem improved their daily life by relieving dystonia. Two patients with cervical dystonia discontinued zolpidem because of poor response and drowsiness.

## Discussion

In this study, zolpidem use significantly improved residual dystonic symptoms after neurosurgical treatment. Thirteen patients (68.4%) showed more than 50% improvement, as measured using the BFMDRS-MS. Drowsiness was the most common adverse event (30%), and no serious adverse events were observed.

The most notable finding of this study was that zolpidem appears to be effective for treating residual dystonia after neurosurgical treatment. Neurosurgical treatments for dystonia are usually considered after failed conservative treatment, including oral medications such as trihexyphenidyl, clonazepam, baclofen, and botulinum toxin injections (1, 2, 20). Unfortunately, the effectiveness of neurosurgical treatment for dystonia varies between patients, and 10 to 25% of patients with cervical, segmental, or generalized dystonia do not respond to selective peripheral denervation or pallidal deep brain stimulation (21–23). Zolpidem can be a treatment option for patients who fail to respond to neurosurgery and conventional conservative treatments.

There have been three studies that evaluated the effects of zolpidem for dystonia improvement using the BFMDRS (4, 10, 12). Among the various phenotypes of dystonia, hand dystonia, evaluated using the ADDS and BFMDRS-MS, showed the most significant improvement, from 26.6% ± 18.7 and 15.2 ± 1.8 at baseline to 61.7% ± 9.9 and 4.8 ± 1.1 after zolpidem intake, respectively. Miyazaki et al. reported improvements in BFMDRS-MS hand scores from 2.9 ± 2.0 before zolpidem administration to 2.0 ± 0.9 after administration of 8.8 ± 5.1 mg zolpidem in eight patients with hand dystonia (10). Five out of eight patients did not respond to zolpidem administration. The effect of zolpidem on dystonia may increase in a dose-dependent manner (10). Pre-treatment BFMDRS values of 58 and 22.5 have been reported in case reports of DYT-6 dystonia and generalized torsion dystonia, respectively. These values improved to 33.5 and 27 with 10 mg and 25.5 and 19 with 20 mg of zolpidem, respectively (12). Our study showed that a single dose of 10 mg zolpidem appeared to be effective in relieving hand dystonia. The relatively lower efficacy of zolpidem for hand dystonia in their study (5) (31.0% improvement in BFMDRS-MS score) compared with our study (68.4% improvement in BFMDRS-MS score) may be due to a lower single dose of zolpidem.

In this study, cases with cervical dystonia responded relatively poorly to zolpidem compared to other regions of dystonia, which nearly corresponded to that in a previous study of seven patients with cervical dystonia who showed a poor response to 10 mg of zolpidem (10). The reason behind the poor response of cervical dystonia to zolpidem is unknown; hence, further studies to determine whether higher doses of zolpidem (≥15 mg) can achieve a better response in patients with cervical dystonia are needed. However, four patients considered that zolpidem obviously improved neck pain measured by the TWSTRS pain scale. Five patients with focal hand or hemidystonia who required a daily dose of 20–30 mg of zolpidem reported zolpidem as a highly effective pain relief medication. Careful evaluation is required for not only objective movement evaluation but also subjective evaluation such as pain and quality of life evaluation.

The mechanism underlying the improvement of dystonia by zolpidem remains unknown. The basal ganglia and thalamus have a high density of GABA-A receptors, which is the binding site of zolpidem (24, 25). In the basal ganglia, the ventral pallidum, substantia nigra pars reticulata, and subthalamic nucleus have the highest density of zolpidem-binding GABA-A receptors, suggesting that zolpidem may help restore the influence of basal ganglia output on the thalamus and motor cortex (26, 27). Badillo et al. suggested that the effects of zolpidem result from the facilitation of inhibitory pathways in the basal ganglia-thalamo-cortical circuit, which leads to the improvement of dystonia (13). However, zolpidem also has therapeutic effects on motor symptoms of Parkinson's disease, which is a hypokinetic movement disorder (28–30). A double-blinded, placebo-controlled study demonstrated that a single oral dose (10 mg) of zolpidem reduced 30.2% of Unified Parkinson's Disease Rating Scale in 10 patients with Parkinson's disease 1 h after the administration (28). The confirmed effects of zolpidem in this study included improvement of rigidity, akinesia, bradykinesia, posture, gait, and facial expression, indicating that zolpidem could serve as a pharmacological equivalent of posteroventral pallidotomy (28). We hypothesize that the underlying mechanism of zolpidem in Parkinson's disease involves the selective inhibition of GABAergic inhibitory neurons in the abnormally overactivated globus pallidus internus (GPi) and substantia nigra pars reticulata, both of which have a high density of zolpidem-binding sites (28). Supposing our hypothesis regarding this mechanism is correct, the suggested mechanism of zolpidem in Parkinson's disease cannot explain the effects of zolpidem on dystonia, which causes reduced output from GPi, leading to increased thalamic and cortical activities (31). Similar paradoxical findings have been suggested in terms of the underlying mechanism that GPi-DBS and pallidotomy (GPi ablation) have similar therapeutic effects on both PD (hypokinetic movement disorder) and dystonia (hyperkinetic movement disorder) (32). So far, available evidence cannot explain in detail the mechanism of zolpidem in dystonia and Parkinson's disease.

This study has some limitations. This is an open-label study and does not have a randomized and controlled design. All patients enrolled in this study received neurosurgical treatment; therefore, the improvements in dystonia in this study did not reflect the true effects of zolpidem on dystonia, which implies that further improvements may have been observed if zolpidem was prescribed before surgery or the lack of response may have been caused by the improved dystonia through surgery. Additionally, the effects of zolpidem on the affected body parts were not statistically evaluated due to the small sample size.

In conclusion, this study suggests that zolpidem can be a valuable treatment option for patients with dystonia. Nevertheless, a randomized controlled trial with larger sample size is needed to elucidate the efficacy and safety of zolpidem for dystonia.

## Data Availability Statement

The original contributions presented in the study are included in the article/Supplementary Material, further inquiries can be directed to the corresponding authors.

## Ethics Statement

The studies involving human participants were reviewed and approved by the Ethics Committee of Tokyo Women's Medical University. Written informed consent for participation was not required for this study in accordance with the national legislation and the institutional requirements.

## Author Contributions

SH: conception and design of the study, acquisition and analysis of data, and drafting a significant portion of the manuscript and figures. KK: conception and design of the study and acquisition and analysis of data. HE and MN: acquisition and analysis of data. TK: conception and design of the study. TT: conception and design of the study and acquisition and analysis of data. All authors contributed to the article and approved the submitted version.

## Funding

This work was supported by JSPS KAKENHI Grant Number JP21K09113.

## Conflict of Interest

The authors declare that the research was conducted in the absence of any commercial or financial relationships that could be construed as a potential conflict of interest.

## Publisher's Note

All claims expressed in this article are solely those of the authors and do not necessarily represent those of their affiliated organizations, or those of the publisher, the editors and the reviewers. Any product that may be evaluated in this article, or claim that may be made by its manufacturer, is not guaranteed or endorsed by the publisher.

## Supplementary Material

Supplementary Video 1 Condition of case 4 (spasmodic dystonia) with and without zolpidem administration. Recording of voice from the cell phone after administering 10 mg of zolpidem (speech condition with zolpidem).

Supplementary Video 2 Condition of case 5 (tongue dystonia) with and without zolpidem administration.

Supplementary Video 3 Condition of case 16 (post-stroke focal hand dystonia) with and without zolpidem administration.
